# Occurrence of various pathogenic and opportunistic fungi in skin diseases of domestic animals: a retrospective study

**DOI:** 10.1186/s12917-020-02460-x

**Published:** 2020-07-17

**Authors:** Bożena Dworecka-Kaszak, Małgorzata J. Biegańska, Iwona Dąbrowska

**Affiliations:** Warsaw University of Life Sciences, Faculty of Veterinary Medicine, Ciszewskiego 8, 02-784 Warsaw, Poland

**Keywords:** Animals, Dermatomycosis, Etiological agents, Fungi, *Otitis externa*, Transmission

## Abstract

**Background:**

Fungal infection of animals or humans are common all over the world. Some of microorganisms like fungi, exist on the skin and can be transmitted onto other individuals, other animal species or even humans and cause skin infections. Moreover, they can be the causative agents of severe generalized infections especially in immunocompromised individuals. The study aimed to evaluate the most frequent etiological agents of skin diseases and to compare the prevalence of animal fungal infections in Poland, and to discuss the possibility of transmission to humans in Poland.

**Results:**

The obtained results are culture based**.** The fungi most frequently isolated from group of animals with skin lesions were *Malassezia pachydermatis* (29.14%), and *Candida* yeasts (27.07%), and dermatophytes (23.5%), including *Microsporum canis* as majority of them (59.25%), and *Trichophyton* genus (40.7%), most of them *T. mentagrophytes,* while *Malassezia pachydermatis* represented (80%) of isolates in animals with *otitis externa.* In over 19% of positive fungal cultures obtained from external ear canals *Candida* yeasts, mainly *C. albicans*, were identified.

**Conclusions:**

Dermatomycoses in companion animals are caused by both, mycelial fungi and yeasts. Most frequently isolated were *Malassezia pachydermatis* and *Candida* spp. Dermatophytes (*Trichophyton, Microsporum*), were also cultured, but the total number of these isolates seems to decrease. We have not found *Cryptococcus neoformans* in tested clinical samples.

## Background

Human as well as animal mycoses are common all over the world. Variety of environmental and physiological factors can contribute to the development of those diseases, e.g. the quantity of fungal elements present in the environment and the efficiency of the host immune system [1–4]. The sources of fungi may also differ. The most common route of infection is aerogenic, thus inhalation of fungal spores present in the air, is the frequent way to acquire the fungi. In such cases the infection starts in the respiratory system [2, 3]. Other important site for fungal colonization and development of mycoses is the skin, which is the largest organ of the body and the first of protective barriers [3, 5]. Its’ major functions include protecting the organism from pathogens, preventing loss of moisture and the regulation of body temperature. Considered as an ecosystem, the skin is the basis for microbial communities of natural microbiota, positively influencing the balance between the health and the disease. The hair coat, the scalp and the hairless skin are quite different niches. Studies characterizing the microbiota inhabiting these niches may answer the fundamental question about the breaking points between the normal colonization and the disease [6]. In recent years, many experiments have been conducted to examine the natural skin microbiome of healthy skin in humans and some animals, especially companion animals [6–8]. Other studies were focused on the qualitative and quantitative changes of the microbiota in various skin diseases [9, 10]*.* Meason-Smith et al. [6], have performed the experiments aiming at characterization of fungal microbiota in dogs and evaluating the influence of body site and health status on the mycobiota. They have used next generation sequencing of ITS (Internal Transcribed Spacer) region and showed, that in general, mucosal sites have had reduced fungal diversity when compared to cutaneous mycobiota. But the composition of fungal species and their diversity were more individual features of animal host than were associated with a particular site of the body. The taxonomic analysis revealed, that dominating phylum of fungi was *Ascomycota*, represented in majority by three genera: *Alternaria*, *Cladosporium* and *Epicoccum*, while the predominant genera among the *Basidiomycota*, were *Cryptococcus* and *Malassezia* [6]. The results of our previous investigation of healthy dogs mycobiota are basically compatible with these data [11].

Among the various fungi included in mycobiota [6, 8] there are genera classified as opportunistic microorganisms. However, even if most of the time opportunists are harmless, their virulence strongly depends on the health status of the host [1, 3, 4]. Such species are also the important etiological agents of various disorders in the group of individuals suffering from other diseases, undergoing surgical procedures or receiving prolonged antibiotic treatment and parenteral nutrition [5].

**Fig. 1:**
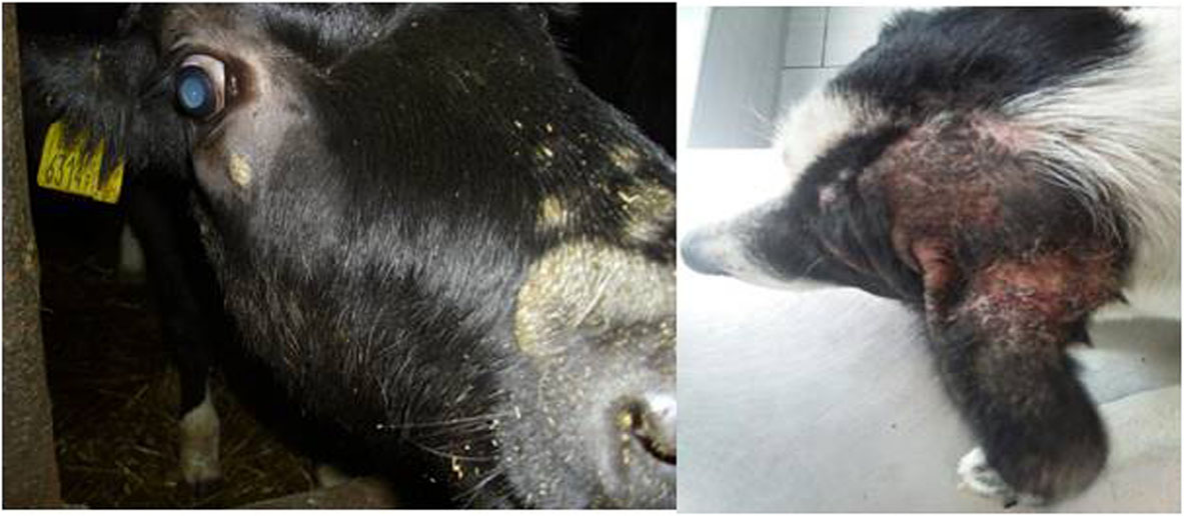
Animal’s dermatomycosis: **a**) Cattle ringworm, **b**) Skin lesions in dogs’ dermatomycosis. (Photographs by I. Kaszak).

Skin diseases, including various mycoses, are among most common diseases affecting mammals [12–16]. In daily veterinary practice, dermatoses of companion animals are also one of the most frequent reasons for visiting the clinic (Fig. 1a, b). Quite frequently the etiological agents are mycelial fungi such as dermatophytes and some moulds as *Alternaria* spp. or *Scopulariopsis* spp. together with yeasts and yeast-like fungi. Skin lesions can be also associated with infections caused by dimorphic fungi, such as *Histoplasma capsulatum*, *Blastomyces dermatitidis* or *Cryptococcus neoformans*, which are known to be pathogenic for both humans and animals, but usually occur in specific geographic regions [17–20]. In some cases, localized fungal colonization of skin, especially with opportunistic yeasts, may initiate persistent mycoses or can be the source of severe systemic fungal infections. The risk of fungal infection and the development of mycoses increases notably in individuals, whose immunity is weakened by other infections (viral or bacterial), parasitic infestations, metabolic diseases, neoplasms or inherited immune disorders [21, 22]. Moreover, most of dermatophytes are able, as true pathogens, to impose the natural barriers of resistance and to spread to many individuals, causing the disease [15].

**The aim of this study** was to assess the occurrence of fungal skin infections in animals in the years 2008–2018 in Poland, to analyse the most prevalent fungal species involved and to evaluate the risk of their transmission to humans.

## Results

The results of bacteriological examination were not analysed for the purposes of this publication. The percentage of positive mycological cultures was lower in animals with dermatitis than in animals with *otitis externa* - 38.18% vs. 71.42%, respectively.

**Fig. 2:**
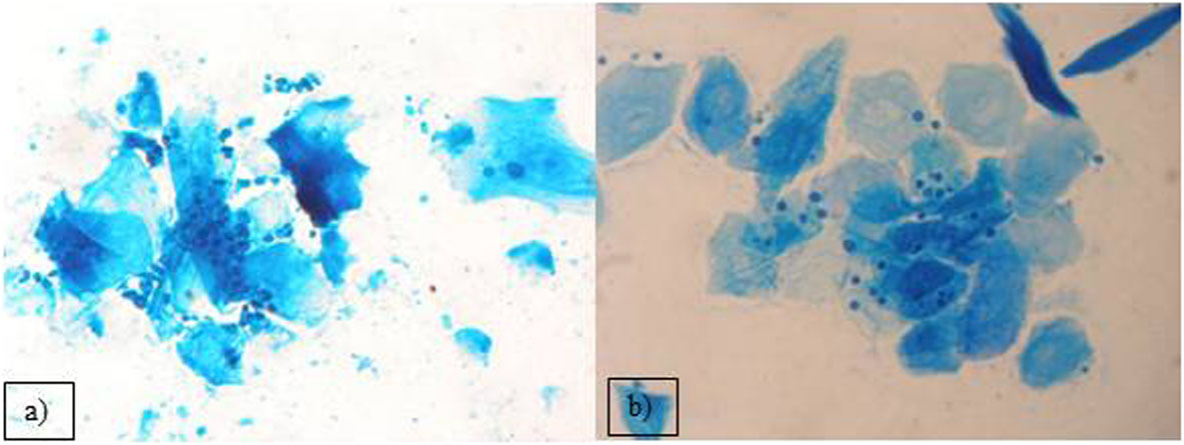
Blastospores of: **a**) *Malassezia pachydermatis* and **b**) *Malassezia globosa* in direct smears from two cases of *otitis externa* in dogs. Methylene blue staining; light microscope × 1000.

**Fig. 3:**
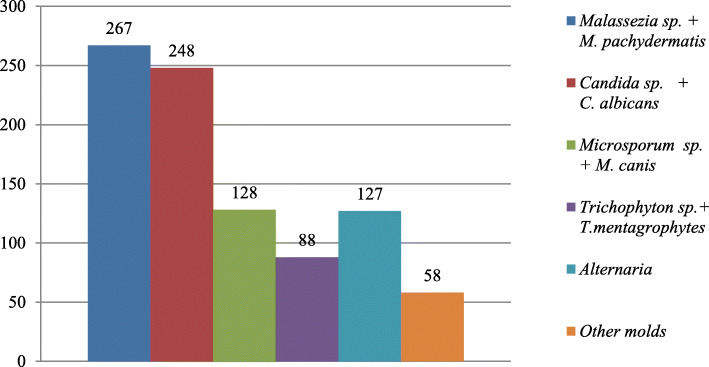
Mycological investigations of samples collected from animals with dermatitis. Colour bars represent indicated fungi and the number of isolates

**Fig. 4:**
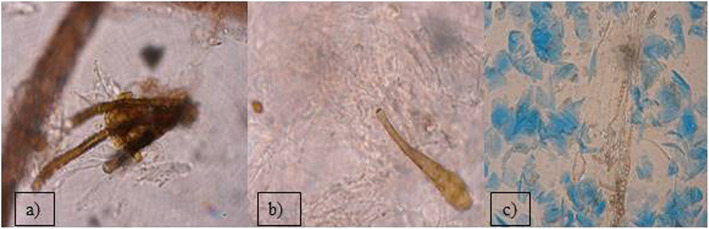
*Alternaria* sp. in *stratum corneum* of horse’s skin: **a**) & **b**) proliferating spores, **c**) chains of spores in horse epithelium. Slides prepared from skin scrapings: **a**) and **b**) Wet mounts; light microscope 400x; **c**) lactophenol cotton blue staining; light microscope 400x.

Out of the 2399 investigated cases of dermatitis, more than half of examined samples (61.8%), were negative for fungal isolation. From the 916 (38.18%), positive cultures, the genus *Malassezia* (267 isolates), mainly *M. pachydermatis* (Fig. 2a) and *Candida* genus (248 cases), mainly *C. albicans*, were the most frequent among yeast-like fungi, 29.14 and 27.07% respectively. In 23.5% cases (216 isolates), dermatophytes were diagnosed, with 128 strains of *Microsporum* spp. and the majority of them (59.25%) were identified as *M. canis.* The other 88 isolates (40.7%), of dermatophytes belonged to the *Trichophyton* genus and most of them were *T. mentagrophytes* (Fig. 3). Other mycelial fungi were also isolated, such as 127 strains of *Alternaria* spp*.* (13.86% of positive cultures), and 58 other mycelial fungi (6.33% of positive cultures, *Aspergillus, Paeciliomyces* or *Penicilium*) (Fig. 3), included to the group of “Molds” for this study. During presented study the examination of clinical materials revealed the presence of *Alternaria* moulds in 127 samples, sometimes as the only isolated fungi. All of these isolates were counted separately from other moulds because of the presence of hyphae and typical spores in skin scrapings (especially in samples from horses) (Fig .4a, b, c.). Moreover, during the direct microscopic examination of hair samples, typical, proliferating poroconidia of *Alternaria* were seen together with hyphae fixed around the hair.

From all 2936 samples collected from animals suffering from *otitis externa*, there were 2097 cultures (71.4%) positive for fungi. The most frequently isolated fungi was also *Malassezia pachydermatis* (1682 cases; 80.2%) (Fig. 2a). In several cases, during the microscopic investigation we have noticed the presence of typical for *M. globosa* round, budding cells (Fig. 2b), but the attempts to grow these yeasts were unsuccessful. Routinely, for isolation of yeast the Sabouraud medium without supplementation of fatty acid was used, so only the lipid-independent strains of *Malassezia* were able to grow. In the group of animals suffering from *otitis externa* the frequency of *Candida* yeasts isolation of was significantly lower – they were grown just in 415 cases (19.8%). Among *Candida* strains we have found mainly *C. albicans*, but also other species like *C. krusei*, *C. glabrata* and *C. parapsilosis* were infrequently isolated.

Pathogenic dimorphic fungi as *Blastomyces dermatitidis* or *Histoplasma capsulatum* have not been found in specimens collected during the ten-year period.

## Discussion

Our retrospective analysis was focused on the specimens from animals with clinical signs of skin diseases (*otitis externa* and various forms of dermatitis), and showed, that in 56.47% of examined animals, fungi belonging to different morphological groups were isolated. We have found basidiomycetous and ascomycetous fungi including *Malassezia* and *Candida* yeasts, opportunistic moulds e.g. *Alternaria*, as well as typical dermatophytes. In the group of animals suffering from dermatitis, yeasts were discovered in 56.2% of positive cultures (*Malassezia pachydermatis* in 29.1%, *Candida* spp. in 27.1%, respectively), while dermatophytes were the etiological agents of skin disease in 23.5% of cases. The carriers of dermatophytes are cats and other companion animals such as rabbits, guinea pigs or dogs [15, 16]. Moreover, the animal’s equipment, like brushes, collars, mattresses or resting places for dogs and cats can be the source of infection, because the spores of dermatophytes are highly resistant in the environment for long period of time. Among all skin mycoses, dermatophytoses are zoonoses and may be transmitted from animals to humans. Clinical cases of dermatophytosis occurring simultaneously in animals and its owner were described in literature, e.g. *Trichophyton rubrum* infection coexisting in a human and in a pig [23], and in a dog [24], or microsporosis transmitted from cat to children [14].

**Fig. 5:**
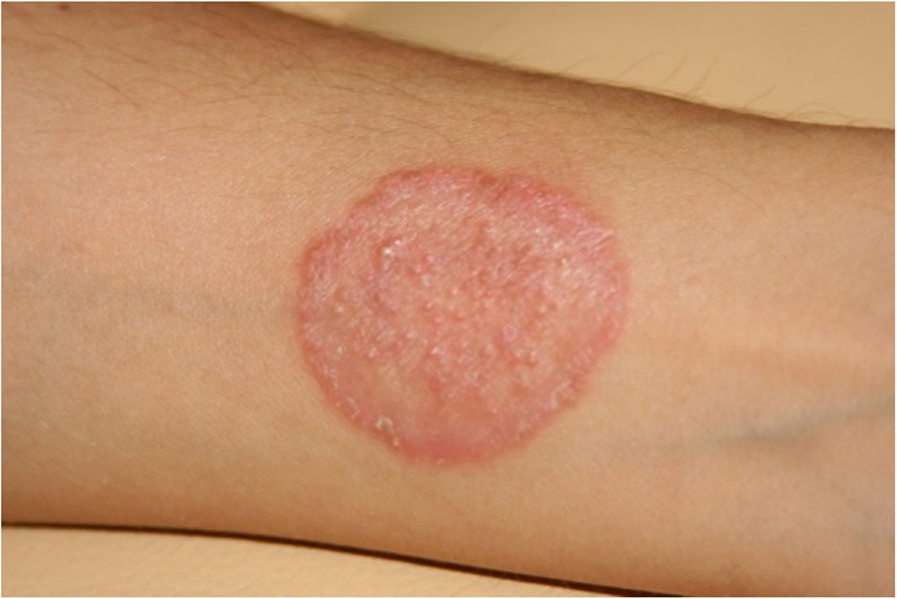
*Tinea manuum* contracted by laboratory personnel.

Additionally, one of those who have conducted this investigation, had experienced dermatophytosis after contact with the infected animals during sampling and with the clinical specimens during repeated diagnostic mycological procedures (Fig. 5.). In this case *Arthroderma benhamiae* (the anamorph of *Trichophyton mentagrophytes*), was cultured from skin scrapings (data not published). Also, we had noticed skin lesions typical for dermatophytosis on the forearm of a woman owning the pet rabbit, from which we had isolated *Microsporum canis*. These findings complies with the literature data from several years describing *Trichophyton mentagrophytes* and *Microsporum canis* infections as the most common etiological agents of human dermatomycosis in Poland [25–27]. Among the *Trichophyton* genus, in few cases we have isolated geophilic *Trichophyton terrestre* (*Arthroderma insingulare*) too. In our former study, focused on mycological screening, we didn’t observe the presence of the dermatophytes’ spores in the environments of healthy animals, their collars or dens [11], however we cannot exclude these sources.

During our investigations we have cultured various moulds such as *Aspergillus, Penicilium* or *Paeciliomyces*. However, while culturing animals hair samples or skin swabs, the growth of such environmental moulds should be treated as the contaminations, because their multiple spores are present in the air, especially in the stables, barns and animal rooms. They are widely distributed in the environment and deposited on the hair coat and skin surface of animals [28].

During the period of 10 years, among the mycelial fungi isolated from cases of cutaneous infections was also *Alternaria* spp*.* In many laboratories, growth of *Alternaria* colonies is treated as typical culture contamination with saprophytic fungi, because they are widely distributed in the environment and colonizing various plants or causing their diseases [29]. *Alternaria* spores are present in the air, soil and water or on the surface of human and animal skin [6, 11, 29]. However, in veterinary medicine, infections due to moulds such as *Alternaria alternata*, especially in horses, as well as hypersensitivities noted frequently in pet animals, have become an emerging clinical problem. Despite *Alternaria* does not belong to keratinolytic fungi, proliferating fungal elements present in the tissues suggest, that it may be responsible for skin damage. Considering the increasing number of cases in which this fungus was found as the only agent in skin lesions [30], it should be noticed that the growing *mycelium* may mechanically damage the neighbouring tissues. In our practice, we have isolated *Alternaria* fungi in 13.8% of positive cultures and we have seen in microscopic slides prepared directly from skin scrapings, that poroconidia of *Alternaria* were able to germinate and produce *hyphae* in *stratum corneum.* In Poland, such reports were sparse until 2013, when Dworecka-Kaszak [31] has described spores of *Alternaria* proliferating in the skin scrapings and has isolated it as the sole causative agent of fungal *dermatitis* in horse. In the same year, Tyczkowska-Sieroń and Głowacka [32], have described dermatomycoses in two Shetland ponies and Beagle dog, in which *Alternaria tenuissima* was the etiological agent. In the international veterinary literature cutaneous fungal granulomas occurring in horses have been described for years. Many authors have noted the cases of equine dermatitis, in which *Alternaria* fungi were found in samples obtained by biopsy of skin nodules [30, 33, 34]. In some clinical samples of this study *Alternaria* was isolated from as the only infectious agent, causing skin lesions characteristic for dermatomycoses, as reported before [30]. *Alternaria alternata* has been recorded as a common indoor allergen, causing different hypersensitivity reactions in humans, sometimes leading to asthma [29, 35, 36]. Although, serious infections in the immunocompetent hosts are rare. This fungus may cause opportunistic infections in immunocompromised patients, especially in those undergoing solid organ or bone marrow transplantation [36]. Fungi of *Alternaria* spp. were also noted as the causative agents of ocular mycosis and onychomycosis connected either with the steroids therapy or post traumatic disorders [36]. Additionally, their presence in the foodstuff (e.g. in crops, vegetables, fruits) is very common.

Our analysis of specimens obtained from animals with clinical symptoms of dermatitis did not reveal cases of infections caused by dimorphic fungi, as these obligate pathogens are endemic for other geographic regions. Even though, there were clinical cases of histoplasmosis reported in European countries (Austria, Italy, Turkey) [37, 38] or cryptococcosis [20], presented retrospective study has confirmed, that they are usually not found in companion animals in Poland.

Beside mycelial fungi, we have also reported the isolation of the genus *Malassezia.* These yeasts are found in different skin regions of healthy human skin [8], and are also present on heathy skin of dogs [6]. Traditionally, the lipid-dependent species were thought to occur only on human skin, while *M. pachydermatis* was assumed to be restricted to animals skin and in particular carnivores [39]. It is suspected, that other *Malassezia* species, like *M. globosa* or *M. sympodialis* or even lipid-dependent strains of *M. pachydermatis* could be involved in developing of *otitis* in dogs, but we were not able to cultivate them [40, 41]. Mentioned species were also isolated from the skin of healthy cats [39, 42]. Although most of the time these yeasts are opportunistic in nature, it may become pathogenic with any alteration in the skin microbiota, disorders of the host defence mechanisms or the imbalance of homeostasis [39, 43–45]. Also dermatitis caused by flea bites, especially when accompanied by hypersensitivity reaction, together with food hypersensitivity or atopy, as well as antimicrobial or corticosteroid therapy, may facilitate proliferation of these yeasts [39]. Additionally there are some canine breeds with higher risk of developing malasseziosis e.g. West Highland White Terriers, Basset Hounds, Beagles, Springer and Cocker Spaniels or Boxers [39, 43–45]. In cats, *Malassezia* infections are more limited and usually are associated with other underlying or concurrent disease such as retro-viral infections [39]. *Malassezia* yeasts, especially *M. pachydermatis*, were also isolated from wild pinnipeds and ferrets, foxes, bears or other animal species such as pigs, horses and also from birds. They were also noted in literature as the etiological agents of infections of such “exotic” host species as rhinoceroses, dromedaries, okapis or elephants [39]. *M. pachydermatis* are still the most common fungal agent contributing to *otitis externa* and other types of dermatitis in domestic animals, particularly in dogs, what has been described by others and confirmed in this report [39, 44, 46–48]. We have isolated this species from over 80% of positive fungal cultures of ear swabs taken from the *otitis externa* cases. Our observations of fungal diversity in clinical specimens obtained from the dogs with various cutaneous hypersensitivities are also congenial with the experiments of Meason-Smith and her group [6]. They have showed, that *Malassezia* colonization of ear canals of allergic dogs were significantly more abundant, than in healthy individuals [6]. Fig.2a and b, have shown the direct microscopic slides prepared from the samples taken from external ear canal of two different dogs suffering from *otitis externa.* In one dog, as well as in many others, the most frequent etiological agent - *M. pachydermatis -* was identified, while in second individual we found *M. globosa* blastospores, what was consistent with the literature data showing the occurrence of this species in the ear canal of dogs. These opportunistic yeasts are of increasing importance in human and animal diseases [8, 9, 49, 50]. *Malassezia pachydermatis* has been described as the cause of sporadic bloodstream infections (BSI), especially in neonates with low birth weight and in premature infants. Most cases of fungal BSI are noted in patients with any intravenous catheters. Other factors increasing the risk of BSI is mechanical ventilation and steroid and antibiotic treatment [49]. Cases of life-threatening fungemia in humans have been attributed to *Malassezia pachydermatis*, for which dogs are a natural host. In some cases, the sources of human infections have been traced to pet dogs owned by healthcare workers [46, 47].

Some *Candida* yeasts, especially *C. albicans*, are endogenic saprophytes, but their role as the opportunistic pathogen is also known and described. Together with *Cryptococcus neoformans* these yeasts are one of the most important etiological agents of opportunistic systemic mycoses especially in immunocompromised patients, who would like otherwise not be infected.

Examples of medical conditions connected with immunosuppression include AIDS, alteration or translocation of normal microbiota caused by antibiotics, immunosuppressive therapy and metastatic cancer or severe surgical procedures. In companion animals, the incidence of *Candida* systemic mycoses is not so high, but the role of these yeasts as causative agent of other mycoses has increased. The medical conditions increasing the susceptibility of animals such as dogs or cats for candydosis are briefly the same as in humans, including diabetes, other endocrinal disorders and neoplastic diseases [21, 51]. There are also suggestions, that these yeasts may play a role in atopic dermatitis (AD) in humans by influencing the production of IgE, but it is still not thoroughly explained or proved [9].

In our Mycology lab. *Candida* yeasts are isolated frequently, mostly from mucous membranes of various animals [21]. We have also observed their growth from skin swabs and scrapings or from ear canal swabs. During reported 10-year period, we have identified *Candida* strains, mainly *C. albicans* in 27.1% of positive fungal cultures in *dermatitis* cases and 19.8% of positive fungal cultures in *otitis externa* cases. Among others, *C. krusei*, *C. glabrata* or *C. parapsilosis*, were isolated too. In this report, we have also shown that the number of *Candida* isolates obtained from companion animals maybe comparable or even similar with the number of *Malassezia* isolates. Our findings may indicate, that fungi from *Candida* genus are important factors of dermatitis in animals, including immunocompetent individuals.

## Conclusions

- Dermatomycoses in companion animals are caused by both mycelial fungi and yeasts.
- Among the animals with dermatitic lesions, yeasts and yeast-like fungi from *Malassezia* and *Candida* genera, were the most frequent fungal etiological agents.
- In more than 20% of positive cultures, dermatophytes were identified, mostly belonging to *Microsporum* genus, while *Trichophyton* were less common.
- We have noted the increasing tendency of *Alternaria* spp. isolation from clinical cases of dermatitis. These fungi should be considered as the causative agents of skin mycoses, basing on the presence of their proliferating spores in the skin samples as the sole organism.
- *Malassezia pachydermatis* was isolated in over 80% of *otitis externa* cases in dogs and remains the most frequent etiological agent of this disease. In this group of animals *Candida* yeasts were revealed in less than 20% of cases, what makes them the second causative agent.
- During the 10 year period there were no clinical cases of dermatomycosis caused by *Histoplasma capsulatum* or *Cryptococcus neoformans* in the region of Mazovia, Poland*.*

## Methods

A total of 5335 specimens, such as hair, skin scrapings, skin or ear swabs were investigated during a 10 years period 2007 to 2016 for fungal infection at the Microbiology Lab from Department of Preclinical Sciences, Faculty of Veterinary Medicine, Warsaw University of Life Sciences-SGGW, Poland. The clinical specimens were obtained from 4150 dogs, 689 cats, 88 rodents and 274 riding horses, 11 birds (e.g. parrots, decorative pigeons), and 123 other pet animals (e.g. reptiles and mammals), and divided in two groups; 2399 from animals with dermatitis and 2936 from animals with *otitis externa*. The group of animals with *otitis externa* was consisted exclusively of dogs and cats. All clinical samples were tested routinely by microscopic examination of direct slide and culturing on Sabouraud Medium (with and/or without cycloheximide). Results were calculated basing on the cultures. All fungal isolates were divided according to their morphology in the group of mycelial fungi (including moulds and dermatophytes), or yeasts and yeast-like fungi. Identification of mycelial fungi was based on the morphology of their colonies and micromorphology of spore and hyphae. Additional techniques, such as Riddel’s microculture or hair perforation test, were also applied. In some cases, for identification of chosen dermatophytes strains, genetic analyses were used. For all of the yeast isolates biochemical and physiological properties were analysed with API Candida and/or ID32C microtests (BioMerieux, France) urease and germ tube tests.

## Data Availability

The datasets used and/or analysed during the current study are available from the authors on reasonable request.

## Abbreviations

ITS: Internal transcribed spacer
AIDS: Acquired immunodeficiency syndrome
KOH + DMSO: Potassium hydroxide and dimethylsulphoxide used for Wet Mount technique, a procedure in which potassium hydroxide is used to dissolve keratin in skin and reveal fungal elements under the microscope
BSI: Bloodstream infections
AD: Atopic dermatitis
